# Effect of Electrohydrodynamic (EHD) on Drying Kinetics and Quality Characteristics of Shiitake Mushroom

**DOI:** 10.3390/foods11091303

**Published:** 2022-04-29

**Authors:** Anjin Xiao, Changjiang Ding

**Affiliations:** College of Science, Inner Mongolia University of Technology, Hohhot 010051, China; 17604786287@163.com

**Keywords:** electrohydrodynamic drying, Shiitake mushroom, drying rate, shrinkage, rehydration, SEM, FTIR

## Abstract

The effect of an electrohydrodynamic (EHD) drying system on the drying kinetics, microstructure and nutritional composition of shiitake mushrooms was studied. Shiitake mushroom slices were dried at 0, 18, 22, 26, 30 and 34 kV. The results showed that the drying rate, effective moisture diffusion coefficient and shrinkage of the EHD treatment group were significantly higher than those of the control group. The 34 kV treatment group had the highest drying rate (0.24 g W/g DM × h) and the highest effective moisture diffusion coefficient (1.01 × 10^−10^ m^2^/s), which were 6.75 and 7.41 times higher than those of the control group, respectively. The control group had the highest rehydration ratio (7.72) and showed unsatisfactory color performance. The scanning electron microscopy (SEM) results showed that compared with the control group, the surface of samples dried by EHD exhibited different degrees of encrustation, and the area of encrustation increased with increasing voltage. After analysis by Fourier transform infrared (FTIR) spectroscopy, it was found that the samples of both the EHD-treated and control groups had similar absorption peak positions, but the intensity of the absorption peak of the EHD-dried samples was greater. Compared with the control group, the shiitake mushroom slices dried by EHD had a higher protein content and polysaccharide content. The polysaccharide content in 22 kV treatment group was the highest (4.67 g/100 g), and the protein content in 26 kV and 34 kV treatment groups was the highest (17.0 g/100 g). This study provides an experimental and theoretical basis for an in-depth study of the drying kinetics of shiitake mushrooms and provides theoretical guidance and clues for the wider application of EHD drying technology.

## 1. Introduction

Shiitake mushrooms (*Lentinus edodes*) are the second largest edible fungus worldwide, accounting for 25% of global fungus production, and the production is increasing faster than that of any other fungus [1]. Shiitake mushrooms contain 18 amino acids, providing an ideal proportion of almost all essential amino acids required for human nutrition. In addition, Shiitake mushrooms are rich in the vitamin B group, vitamin C, ergosterol, phytosterols, minerals, lentinan and so on [2,3]. Shiitake mushrooms have very important medicinal value, with antitumor and anticancer effects, and can help reduce blood lipids and enhance human immunity [4,5,6]. However, the water content of fresh shiitake mushrooms is very high, generally 85–95%, with a very short shelf life. If these mushrooms are not treated in time, they will easily deteriorate.

Drying is the most common processing method for preserving shiitake mushrooms, which can greatly prolong the shelf life of shiitake mushrooms, and dried shiitake mushrooms have unique flavors [7]. Most of the traditional drying methods are thermal drying, such as hot air drying, microwave drying and infrared drying. These methods generally have a negative impact on the color, shrinkage, and nutrient content of dried products [8,9]. In recent years, many researchers have explored the effects of different drying methods on shiitake mushrooms. In 2014, Qi et al. used vacuum microwave jet drying, hot air drying and mid-infrared drying to dry shiitake mushroom respectively, and found that vacuum microwave jet drying performed best in sensory evaluation, texture, color and total sugar content [7]. In 2014, Wang et al. compared the results of three mixed drying techniques on the drying characteristics and quality parameters of shiitake mushrooms, and the results showed that the drying time of hot air combined with microwave drying was the shortest, and the color properties and nutrition retention of hot air combined with radiofrequency drying and mid-infrared assisted convection were better [10]. In 2021, Karim et al. applied cold plasma pretreatment to hot air drying of shiitake mushrooms, and the results showed that cold plasma induced surface modification of fresh shiitake mushrooms, accelerated the drying rate and retained higher nutritional properties [11]. In 2011, Motevali et al. used various drying methods to evaluate the energy consumption of shiitake mushroom slice drying, including microwave, infrared, hot air, vacuum, hot air-infrared and microwave-vacuum methods, and the results showed that the energy consumption of vacuum drying was the highest, and that of hot air infrared drying was the lowest [12]. Although these studies are very detailed in some respects, they are basically in the laboratory stage and have not entered the field of large-scale industrial production. Thus, it is necessary to explore new drying technology for shiitake mushrooms.

Electrohydrodynamic (EHD) drying is a new nonthermal and energy-saving drying technology that is particularly suitable for heat-sensitive food and biological products, which has advantages over other drying methods and is becoming a research hotspot [13,14,15,16]. Compared to thermally dried foods, EHD-dried foods have much lower shrinkage, color degradation and texture changes. In addition, EHD-dried foods have a lower loss of soluble solids, higher rehydration ratio and better preservation of nutrients and sensory properties [17,18]. Bashkir et al. tested the effect of different types and arrangements of emitters on the drying effect [19]. Elmizadeh et al. investigated the effects of EHD drying and hot air drying on the effective antioxidant activity and phenolic compounds in quince, and the results showed that the antioxidant capacity and total phenolic compounds of hot air dried quince slices were 1.15 and 1.37 times higher than those of the EHD method, and the average energy consumption of hot air drying was 48.66 times higher than that of EHD drying [20]. Ni et al. investigated the effects of different pretreatment methods on the drying characteristics and surface microstructure of goji berries during EHD drying [21]. Ding et al. compared the effects of EHD drying and oven drying on carrot slices, and the results showed that carrot samples dried by EHD had a higher rehydration rate and carotene content [22]. Martynenko et al. applied EHD drying to apple slice drying and studied the effects of EHD on drying kinetics, energy consumption and color change [23]. Polat et al. applied EHD drying to apricot. The results showed that EHD could better retain the microstructure of the sample than hot air drying, and no cracks appeared on the surface [24]. Dinani et al. investigated the effect of EHD drying combined with hot air drying on the drying characteristics, energy consumption and effective moisture diffusion coefficient of shiitake mushroom slices [25]. In recent years, EHD technology has also been used in the processing of marine products. Bai et al. applied EHD drying to the drying of sea cucumber. Compared with natural drying and oven drying, EHD showed better performance in the rehydration ratio, shrinkage, acid mucopolysaccharide content and protein content of samples [26]. Tamarit-Pino et al. applied EHD to drying Chilean sea cucumber and obtained better drying parameters [27].

Material drying is a very complex process, and the effects are completely different with different materials. Although the application of EHD drying in various materials has been reported in detail, there is a lack of systematic research on the drying characteristics and quality characteristics of shiitake mushrooms by EHD drying. Most studies on EHD drying of shiitake mushrooms mainly focus on physical parameters, such as the drying rate, color, shrinkage and rehydration ratio, but the microstructure and nutritional composition of dried samples have not been fully studied. At the same time, the existing reports did not fully explain the reasons for the changes in samples after EHD drying. In this paper, we used EHD drying to dry fresh shiitake mushroom slices and measured the drying rate, effective moisture diffusion coefficient, shrinkage, rehydration ratio and surface color difference of mushroom slices at different voltages. The effects of EHD drying on the surface microstructure and chemical composition of shiitake mushrooms were investigated by scanning electron microscopy (SEM) and Fourier transform infrared (FTIR) spectroscopy, and the polysaccharide and protein contents of shiitake mushrooms were measured after drying at different voltages. This work provides a theoretical basis for the wide application of EHD in the field of shiitake mushroom drying.

## 2. Materials and Methods

### 2.1. Sample Preparation and Processing

Fresh shiitake mushrooms (*Lentinus edodes*) were purchased from the supermarket near the Inner Mongolia University of Technology. Shiitake mushrooms of the same origin that were free of rot and of the same maturity were selected, and the diameter of the cover was 6 ± 1 cm. The mushroom experiment was immediately performed, avoiding water loss due to storage. After removing the stalks, the fresh mushrooms were cleaned, and the surface moisture was removed through drying. The shiitake mushrooms were cut into round pieces with a diameter of 2.5 cm and a thickness of 0.4 cm with a mold (samples were taken from the central part of shiitake mushroom, and one sample was provided for each mushroom).

### 2.2. Experimental Equipment

**Figure 1:**
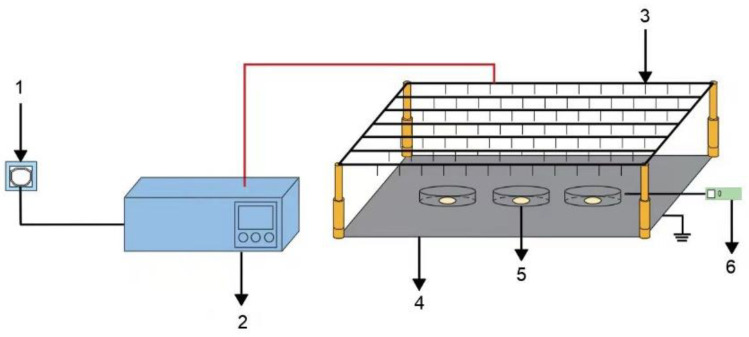
EHD drying system. 1. Power source; 2. High-voltage power supply control system; 3. Needle electrode; 4. Ground electrode; 5. Sample; 6. Anemometer.

This device was composed of a high-voltage power supply control system (YD(JZ)-1.5/50, Wuhan, China) and a multiple needle-to-plate electrode system. The high-voltage power supply control system can output AC voltage or DC voltage, with AC voltage adjusted from 0 to 50 kV and DC voltage adjusted from 0 to 70 kV. AC voltage is used in this experiment. Needle electrodes were connected to the high-voltage power supply control system. The needle electrodes were made of stainless steel and connected to the high-voltage power control system. The needles were 20 mm long with a diameter of 1 mm, and the distance between the two needles was 40 mm. The ground electrode was a 1000 mm × 550 mm stainless steel plate with a distance of 100 mm from the needlepoint (Figure 1).

### 2.3. Experimental Method

The pretreated shiitake mushroom slices were placed in the culture dish and placed in the EHD drying system. Three pieces of shiitake mushroom slices were placed in each experiment and dried after selecting the voltage (0, 18, 22, 26, 30, 34 kV, and 0 kV as control). The mass of shiitake mushroom slices was measured every 0.5 h by an electronic balance (BS124S, Shanghai, China). According to Formulas (3) and (4), the moisture content and drying rate of shiitake mushroom slices were calculated at different times until the moisture content of shiitake mushroom slices was lower than 5% and the drying was stopped.

### 2.4. Initial Moisture Content

The initial moisture content of fresh shiitake mushrooms was determined by a rapid moisture detector (Sh10A, Weifang, China). The initial moisture content of fresh shiitake mushrooms was 9 g/g (dry basis).

### 2.5. Moisture Ratio

The moisture content and moisture ratio of the shiitake mushroom during the drying process are defined as [21]:

(1) $$M_{i}=\frac{m_{i}-m_{g}}{m_{g}}$$

(2) $$\mathrm{MR}=\frac{M_{i}-M_{e}}{M_{0}-M_{e}}$$

where $M_{i}$ is the moisture content (g water/g dry matter) at time $t_{i}$, $m_{g}$ is the dry mass of shiitake mushroom, $m_{i}$ is the mass of the shiitake mushroom at time $t_{i}$, MR is the moisture ratio, $M_{0}$ is the moisture content at time $t_{0}$ and $M_{e}$ is the equilibrium moisture content. Since the equilibrium water content of food materials is generally very small and negligible, the value of *m* is considered to be zero. Therefore, the formula of moisture ratio can be simplified as [28]:

(3) $$\mathrm{MR}=\frac{M_{i}}{M_{0}}$$

### 2.6. Drying Rate

The drying rate of Shiitake mushroom in the drying process is defined as [29]:

(4) $$\mathrm{DR}=\frac{M_{t}-M_{t+∆t}}{∆t}$$

where DR is the drying rate (g water/g dry matter × h), $M_{t}$ is the moisture ratio of the shiitake mushroom at time t and $M_{t+∆t}$ is the moisture ratio of the shiitake mushroom at time t+∆t.

### 2.7. Effective Moisture Diffusion Coefficient ($D_{eff}$)

The effective moisture diffusion coefficient ($D_{eff}$) of shiitake mushrooms during the drying process was calculated using Fick’s second law [30]:

(5) $$\frac{dM}{dt}=D_{eff}\frac{d^{2}M}{dr^{2}}$$

For a long drying process, and the equation can be expressed as [31]:

(6) $$\mathrm{MR}=\frac{8}{\pi^{2}}\exp\left(-\frac{\pi^{2}D_{eff}t}{4L^{2}}\right)$$

where $D_{eff}$ is the effective moisture diffusion coefficient of the shiitake mushroom and L is half the thickness of the sample. This equation can be written as [32]:

(7) $$\ln\left[\mathrm{MR}\right]=-\frac{\pi^{2}D_{eff}}{4L^{2}}t+\ln\left[\;\frac{8}{\pi^{2}}\right]$$

The slope of the above equation is:

(8) $$\mathrm{slope}=-\frac{\pi^{2}D_{eff}}{4L^{2}}$$

$D_{eff}$ can be measured from the plot slope.

### 2.8. Shrinkage

The shrinkage was measured by a displacement method. Glass beads (0.12–0.15 mm) were used as a substitute medium. The shrinkage of shiitake mushroom was calculated as [1]:

(9) $$\mathrm{SR}=\frac{V_{0}-V_{d}}{V_{0}}\times 100\%$$

where SR is the shrinkage of shiitake mushroom, $V_{0}$ is the volume of fresh shiitake mushroom and $V_{d}$ is the volume of dried shiitake mushroom.

### 2.9. Rehydration Ratio

We put 2~3 dried shiitake mushrooms slices into a 150 mL beaker, added 100 mL of deionized water and soaked the beaker in a constant temperature water bath at 30 °C for 1 h. Then, the shiitake mushroom slices were removed, filter paper was used to absorb the excess water on the surface and weighed and the formula for calculating the rehydration ratio was as follows [22]:

(10) $$\mathrm{RR}=\frac{m_{a}}{m_{b}}$$

where RR is the rehydration ratio of shiitake mushroom, $m_{a}$ is the mass of the dried shiitake mushroom after rehydration and $m_{b}$ and $m_{a}$ are the weight of samples before and after rehydration, respectively.

### 2.10. Color

The brightness value ($L^{*}$), redness value ($a^{*}$) and yellowness value ($b^{*}$) of shiitake mushroom before and after drying were measured by an automatic colorimeter (3nh-NR60CP, Shenzhen Threenh Technology Co., Ltd., Shenzhen, China). The instrument was calibrated before the test. During the test, five positions of the same sample were measured and the results were averaged. The total color difference (ΔE) is calculated as [1]:

(11) $$\Delta E=\sqrt{{\left(L_{1}^{*}-L_{0}^{*}\right)}^{2}+{\left(a_{1}^{*}-a_{0}^{*}\right)}^{2}+{\left(b_{1}^{*}-b_{0}^{*}\right)}^{2}}$$

where $L_{0}^{*}$, $a_{0}^{*}$ and $b_{0}^{*}$ are the brightness, redness and yellowness value of the shiitake mushroom sample before drying, respectively, and $L_{1}^{*}$, $a_{1}^{*}$ and $b_{1}^{*}$ are the brightness, redness and yellowness value of mushroom slices after drying, respectively. ΔE represents the total color difference, which refers to the degree of total color change in the dried sample compared with the color of the fresh sample.

### 2.11. Scanning Electron Microscopy (SEM)

A scalpel was used to intercept the middle uniform and flat part of the dried mushroom slices (to ensure that the interception process would not damage the surface of dried mushroom slices), and the intercepted part was attached to the sample carrier table using carbon conductive adhesive, followed by gold spraying. A field scanning electron microscope (SU8020, Tokyo, Japan) was used to scan and photograph the same area of each sample under a condition of accelerating voltage of 20 kV and a magnification of 250 times [11].

### 2.12. FTIR Spectroscopy

The dried shiitake mushroom pieces were ground and mixed with potassium bromide at a ratio of 1:100, and then put into a tablet machine (HY-12, Jingjiang, China) to make slices. The sample was scanned using an FTIR spectrometer. The infrared spectra were recorded from 4000 to 400 cm^−1^ with 32 scans per spectrum at 4 cm^−1^ resolution [33].

### 2.13. Polysaccharide and Protein Content

The polysaccharide content of dried shiitake mushroom slices was determined using the phenol-sulfuric acid method [34]. The protein content was determined by the Kjeldahl method [35]. Three measurements were taken each time, and the average was recorded.

### 2.14. Statistical Analysis

All experiments were conducted in triplicate, and the experimental results are expressed as the means ± SD. Analysis of variance (*p* < 0.05) was used to analyze the data, and Duncan’s multiple range test was used to separate the means.

## 3. Results and Discussion

### 3.1. Moisture Ratio

**Figure 2:**
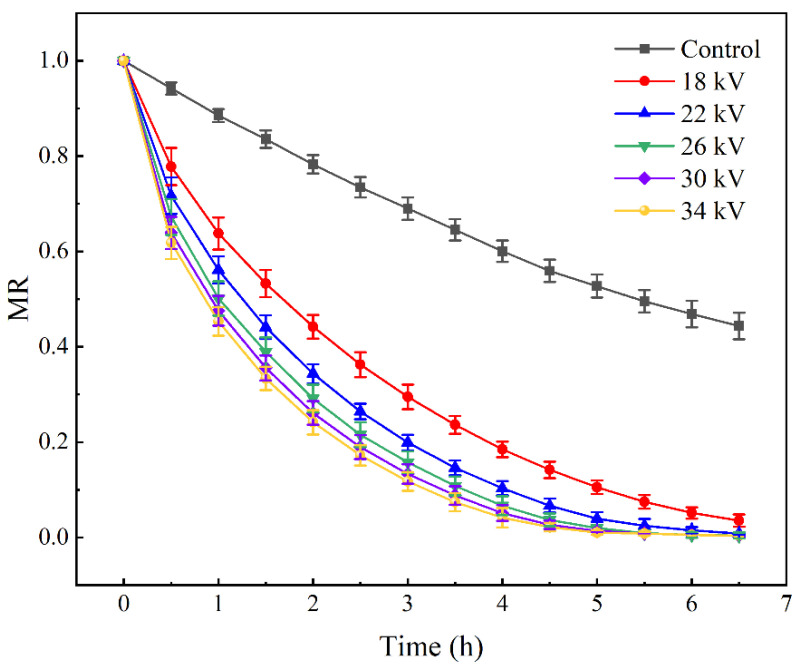
Curve of the moisture content of shiitake mushroom slices.

Figure 2 shows the curve of the moisture ratio of shiitake mushroom slices changing with time at different voltages. The decline rate of the moisture ratio was slowest in the control group and fastest in the 34 kV treatment group. The decline rate of the moisture ratio in the EHD treatment group was higher than that in the control group, and the decline rate increased with the increasing voltage, but the increment gradually decreased. The drying curves of 30 kV and 34 kV were basically coincident. This result is consistent with the conclusions of Ding and Bai et al. [22,36]. Because EHD drying mainly affects the surface of the material, the drying process is basically a deceleration period [37].

### 3.2. Average Drying Rate and Average Drying Time

**Figure 3:**
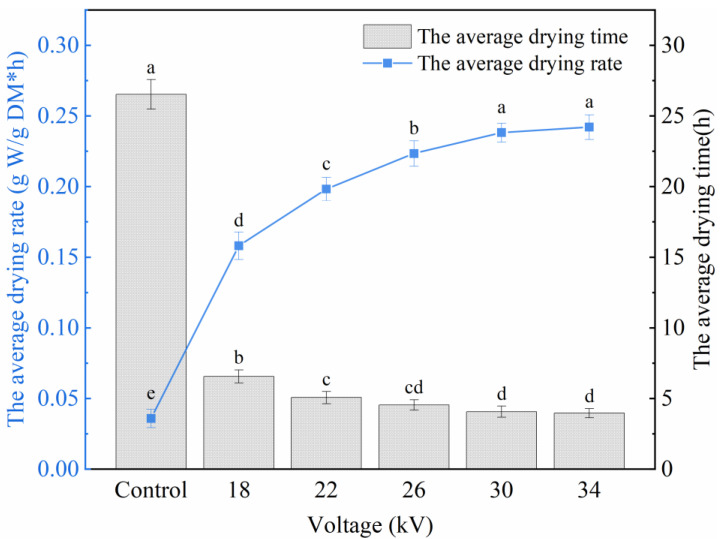
Average drying rate and average drying time of shiitake mushroom slices. Different letters indicate significant differences (*p* < 0.05) between sample means.

Figure 3 shows the average drying rate and average drying time of shiitake mushroom slices at different voltages. The average drying rate of the EHD treatment group was significantly higher than that of the control group (*p* < 0.05). The average drying rates of 18, 22, 26, 30 and 34 kV were 4.41, 5.53, 6.23, 6.64 and 6.75 times that of the control group, respectively. The drying rate increased with increasing voltage, and the increment decreased gradually. This result is consistent with the results of Elmizadeh et al. [20,38].

The effect of voltage on the drying rate can be explained by the increase in ionic wind speed. The ionic wind generated by the tip discharge will impact the material surface and disturb the saturated air layer, resulting in enhanced water evaporation, while the higher voltage will produce stronger ionic wind, thus improving the water evaporation rate [39,40]. In addition, when food is exposed to an electric field, some of its constituent molecules are polarized in the electric field, and the rotation of the dielectric molecule under the action of the electric field will lead to an increase in the mass transfer coefficient [20]. According to the report of Iranshahi et al., in the process of EHD technology applied to the drying of plant-based food, the convective dehydration of ionic wind is the main dehydration mechanism, and the contribution rate to the total water transport of plant capillary-porous materials is about 93% [37].

The average drying time of the EHD treatment group was also significantly shorter than that of the control group (*p* < 0.05), with 75.26%, 80.92%, 82.88%, 84.69% and 85.06% reductions in the average drying time at 18, 22, 26, 30 and 34 kV, respectively, compared to the control group. The drying time of shiitake mushroom slices decreased with increasing voltage. Under different voltage drying conditions, the average drying rate is inversely proportional to the average drying time. The higher the drying rate is, the shorter the drying time.

### 3.3. Effective Moisture Diffusion Coefficient ($D_{eff}$)

**Figure 4:**
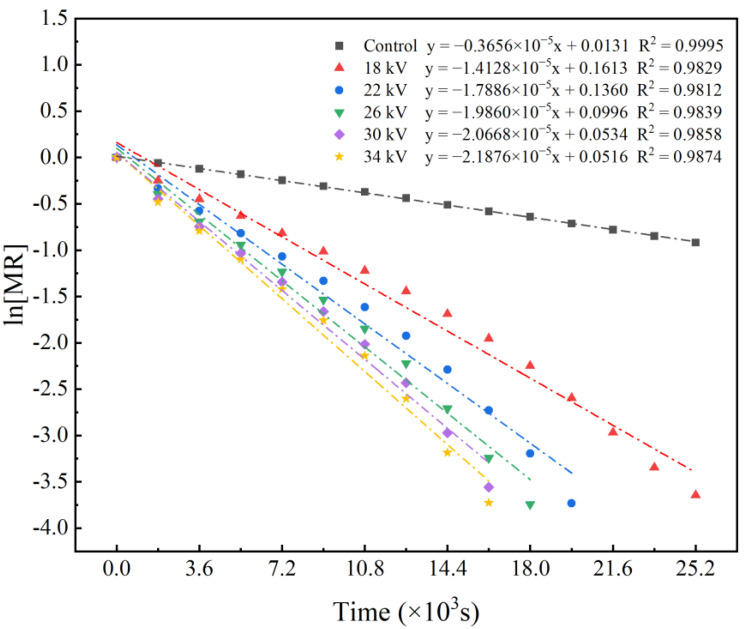
Linear evaluation of ln [MR] over time.

**Figure 5:**
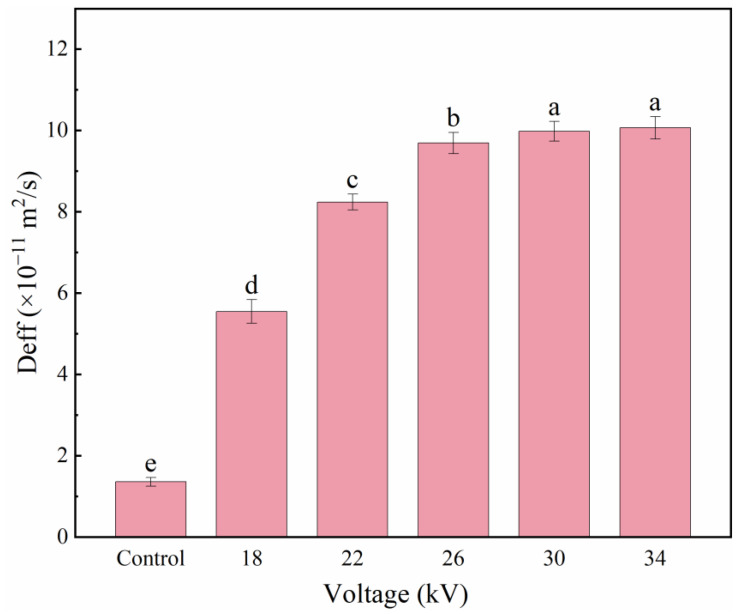
$D_{eff}$ of shiitake mushroom slices. Different letters indicate significant differences (*p* < 0.05) between sample means.

Figure 4 shows the linear evaluation of ln [MR] over time. It can be seen from the figure that the slope of ln [MR] decreases as the voltage increases, indicating that the decline rate of moisture ratio gradually accelerates as the voltage increases. Figure 5 shows the $D_{eff}$ of shiitake mushroom slices at different voltages. The $D_{eff}$ of the control group is the smallest, and the $D_{eff}$ of the 34 kV treatment is the largest. The $D_{eff}$ of each treatment group was significantly higher than that of the control group (*p* < 0.05). Similar to the average drying rate, $D_{eff}$ increases with increasing voltage, and the increment decreases with increasing voltage, which are 4.08, 6.06, 7.13, 7.34 and 7.41 times those of the control group, respectively. This was similar to the results obtained by Martynenko et al., who dried mushrooms with an EHD system, and Ding et al., who dried carrots with an EHD system [22,38]. A higher voltage will produce a stronger ionic wind and improve the water evaporation rate, and thus, have a higher effective $D_{eff}$. Thus, EHD treatment can effectively improve the $D_{eff}$ of shiitake mushroom slices.

### 3.4. Shrinkage

**Figure 6:**
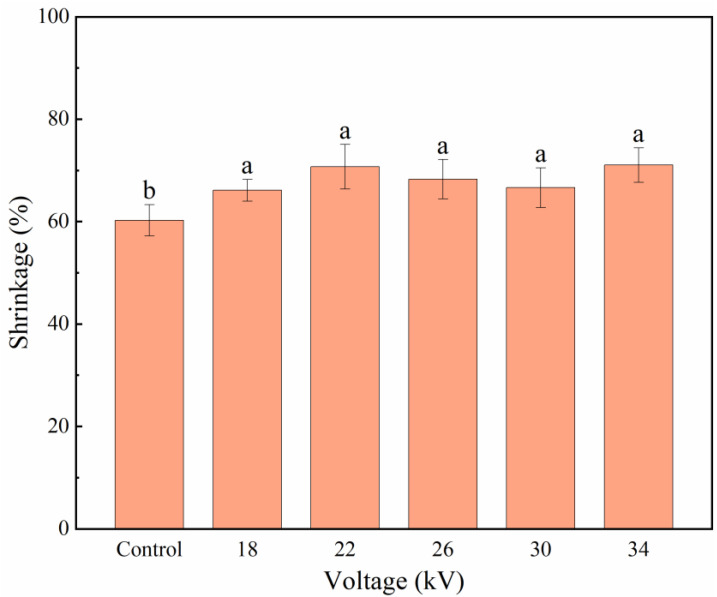
Shrinkage of shiitake mushroom slices. Different letters indicate significant differences (*p* < 0.05) between sample means.

Shrinkage is an important index reflecting the damage degree of fruit and vegetable cell structures, which may affect customers’ preferences [41]. Figure 6 shows the shrinkage of shiitake mushroom slices after different voltage treatments. The shrinkage of the control group was slightly lower than that of the treatment groups (*p* < 0.05), and there was no significant difference in the shrinkage among the treatment groups (*p* > 0.05). There was no obvious linear relationship between voltage and shrinkage. This is consistent with the results of Alemragabi et al. [42].

### 3.5. Rehydration Ratio

**Figure 7:**
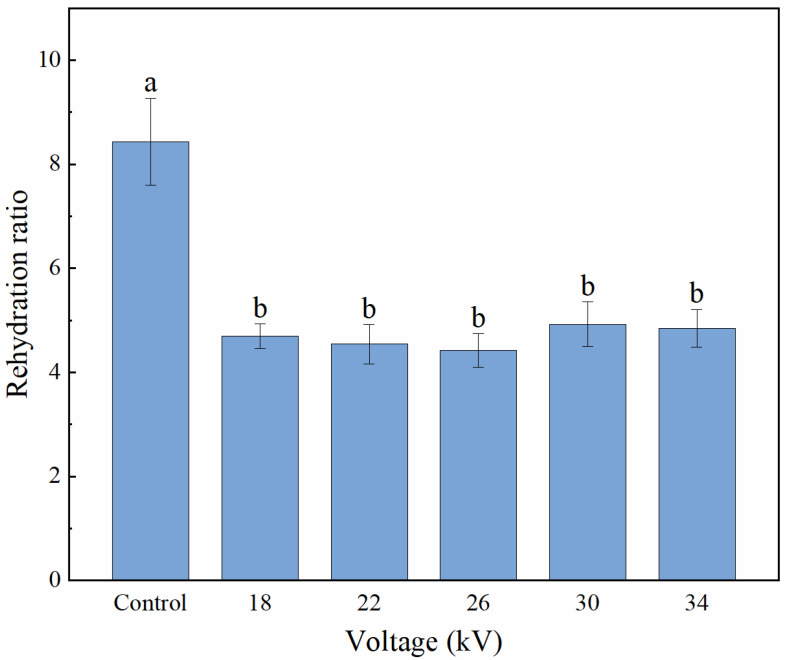
Rehydration ratio of shiitake mushroom slices. Different letters indicate significant differences (*p* < 0.05) between sample means.

The rehydration ratio is the ratio of the weight of dry products after rehydration to the weight of dry products before rehydration, which is considered a representative index to evaluate the damage caused by drying [43]. Figure 7 shows the rehydration ratio of shiitake mushroom slices treated with different voltages. The rehydration ratio of the control group was significantly higher than that of the EHD treatment group (*p* < 0.05), but there was no significant difference in the rehydration ratio among the EHD treatment groups (*p* > 0.05).

The relationship between the rehydration ratio and shrinkage of the materials is very complicated, and there is no obvious consistency. For example, Tian et al. dried shiitake mushrooms using hot air drying (HAD), microwave drying (MD), vacuum drying (VD) and microwave vacuum drying (MVD), respectively, and the results showed that the rehydration ratio was ranked from low to high as MD (293.69%), HAD (335.04%), VD (341.46%) and MVD (398.66%), and the shrinkage ratio was ranked from low to high as MD (40.74%), MVD (45.45%), VD (51.65%) and HAD (54.55%) [1]. Ding et al. used an EHD system to dry Chinese wolfberry fruits under different voltages, and the results showed that the rehydration ratio of the dried samples increased with the increasing voltage, and there was no significant difference in the shrinkage of the samples treated with different voltages [44]. Esehaghbeygi et al. compared banana slices after EHD drying and microwave drying, and the results showed that the average shrinkage of EHD and microwave drying processes were 0.21 and 0.29 m^3^/m^3^, respectively, and that EHD showed a stronger rehydration capacity [45]. The reason for this phenomenon may be that the shrinkage is mainly related to the moisture content of the material before and after drying, while the rehydration rate is mainly related to the choice of drying method.

### 3.6. Color

Color is one of the key quality parameters affecting consumer acceptance and the market value of products. Due to the pigment degradation, enzymatic browning reactions, Maillard reaction, nonenzymatic browning reactions, vitamin C oxidation and other factors, drying treatment may have a negative impact on food color [46,47]. High-quality dried shiitake mushroom products should have higher brightness values and lower color differences [48]. In low-temperature experiments, we predicted that enzymatic browning was the main factor affecting color change [23].

Table 1 gives the surface color parameters of shiitake mushroom slices treated by different voltages. There was no significant difference in $L^{*}$ between the control group and the fresh shiitake mushroom (*p* > 0.05), but there was a significant difference in $L^{*}$ between the EHD treatment groups and the fresh shiitake mushroom (*p* < 0.05), and the $L^{*}$ increased with increasing voltage. There was no significant difference in $a^{*}$ between the control group and the EHD treatment groups (*p* > 0.05), both of which increased compared to the fresh samples. We proposed that the higher $L^{*}$ of the treatment group than that of the control group was due to the presence of flat encrustation on the surface of the dry products, which resulted in enhanced light reflection on the material surface and increased brightness value. Due to enzymatic browning, vitamin C oxidation and other factors, the $a^{*}$ and $b^{*}$ of dried mushrooms will increase compared to fresh mushrooms. Compared with the control group, the EHD treatment group has a smaller $b^{*}$, probably because the faster drying rate makes the moisture evaporate rapidly, leading to a decrease in the rate of enzymatic browning and other oxidation reactions [49,50]. Other papers mentioned that ionic wind impact wet materials will produce tangentially and high shear force, which can be considered the cause of enzyme inactivation [45]. The total color difference value (ΔE) of the EHD treatment group was significantly smaller than that of the control group (*p* < 0.05), and there was no significant difference among the treatment groups (*p* > 0.05), indicated that the color of the samples in the EHD treatment group was closer to that of the fresh samples. In conclusion, compared with the control group, EHD drying has unique advantages in preserving the color of shiitake mushrooms.

### 3.7. Surface Microstructure

**Figure 8:**
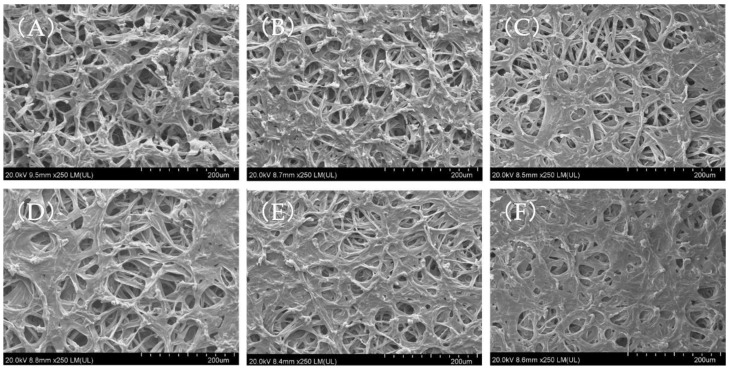
Surface microstructure of shiitake mushroom slices treated by different voltages. (**A**) Control; (**B**) 18 kV; (**C**) 22 kV; (**D**) 26 kV; (**E**) 30 kV; (**F**) 34 kV.

Figure 8 shows the surface microstructure of shiitake mushroom slices treated with different drying voltages. As shown in the figure, the hyphae of the shiitake mushroom slices showed different degrees of contraction and collapse after drying. Compared with the control group, the surface of the samples in the treatment group showed different degrees of encrustation. The encrustation area increased with increasing voltage, and the encrustation area in the 34 kV treatment group was the largest.

We concluded that the faster drying rate in the EHD treatment group led to the contraction and collapse of tubular hyphae, and with the transfer of water, the soluble solids and nonvolatile compounds (such as sugar, minerals and vitamins) exuded from the damaged cells were dragged together with liquid water to the outer surface of the product to form an encrustation [51,52]. At the same time, a relatively high voltage will increase cell membrane electroporation, allowing for more soluble solid and nonvolatile compounds to leak out [37,53]. In addition to the appearance of encrustation on the surface, the original honeycomb structure on the surface of shiitake mushroom slices dried by EHD was compressed into a dense network structure, the hyphae were flat and the gap between the hyphae decreased. The contraction of hyphae in the control group was more obvious, but the gap between hyphae was large and maintained a loose and porous honeycomb structure (Figure 8A). The encrustation and dense structure of the treatment group may be the reason for its large shrinkage and low rehydration ratio.

### 3.8. FTIR Spectroscopy

**Figure 9:**
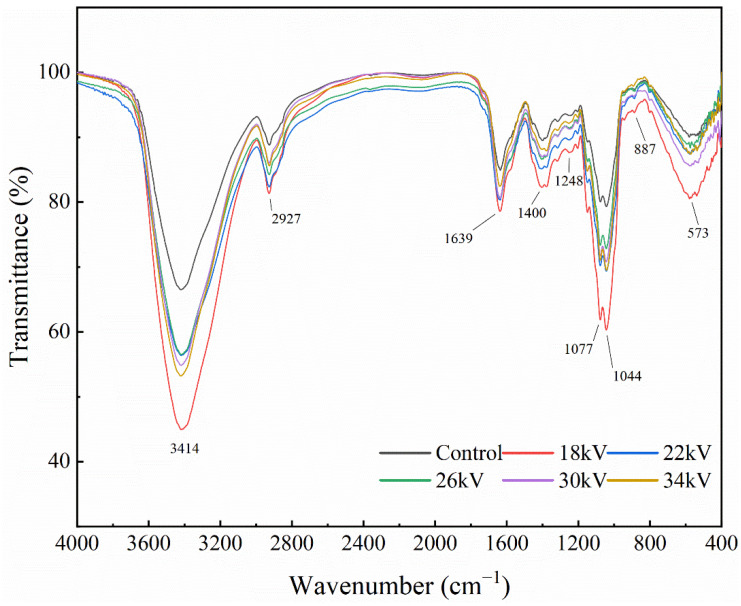
Infrared spectra of shiitake mushroom slices.

The samples of the control group and the treatment group were analyzed by FTIR spectroscopy to determine the effect of EHD drying on the absorption bands of shiitake mushroom samples from 4000 to 400 cm^−1^. As shown in Figure 9, there was almost no change in the position of the absorption peaks of shiitake mushroom slices after different voltage treatments, but the intensity of the absorption peaks was significantly different. The peak intensity of the EHD treatment group was higher than that of the control group. The peak intensity of 18 kV is the largest, and the peak intensities of 22, 26, 30 and 34 kV are similar.

FTIR spectra of dried shiitake mushroom slices displayed strong absorption bands at 1639, 1077 and 1044 cm^−1^, revealing that protein and polysaccharide were the main components [54]. The strong absorption peaks at 3414 cm^−1^ and 2927 cm^−1^ were the characteristic stretching absorption peaks of O–H and C–H, respectively [55]. The typical bands of protein are centered at approximately 1639 cm^−1^ and 1540 cm^−1^, corresponding to the Amide I and Amide II groups, respectively. These bands reflect C=O stretching vibrations with minor contributions from out-of-plane C–N stretching vibrations and N–H bonding and C–H stretching of proteins, respectively [56]. Lentinan is the most important functional component of shiitake mushrooms, producing characteristic FTIR absorption bands at 1639 cm^−1^ (C=O stretching vibration), 1400 cm^−1^ (carboxylate) and 1248 cm^−1^ (C–O–C stretching vibration) [57]. The strong absorption bands at 1077 cm^−1^ and 1044 cm^−1^ are mainly attributed to C–O and C–C stretching vibrations in polysaccharides [57]. The region of 900–400 cm^−1^ is mainly assigned to the existence of polysaccharides, such as β-D-glucan and the pyranose form of glucose [58,59,60,61,62].

In our results, the EHD treatment group and the control group had similar absorption peak positions, which confirmed that the functional groups did not change significantly after EHD drying, and could better retain the nutrients of shiitake mushrooms.

### 3.9. Polysaccharide and Protein Contents

**Figure 10:**
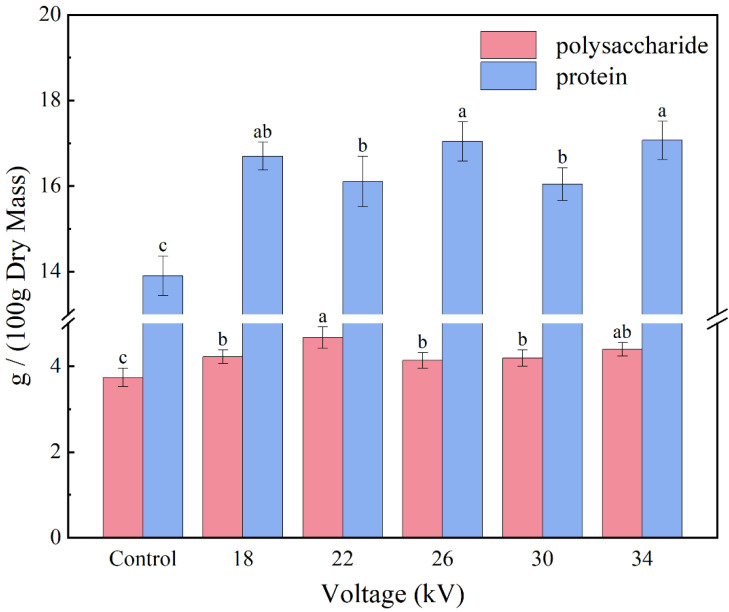
Contents of polysaccharide and protein in shiitake mushroom slices. Different letters indicate significant differences (*p* < 0.05) between sample means.

Lentinan has been recognized as a functional food due to its low toxicity and biological activity, including antioxidant capacity, inhibition of cancer growth and prevention of viral infection [63,64]. Therefore, it is very important to detect the polysaccharide content in dried shiitake mushrooms. Figure 10 shows the polysaccharide and protein contents in shiitake mushroom slices treated with different voltages. The polysaccharide and protein contents in the EHD treatment group were significantly higher than those in the control group (*p* < 0.05), and there was no significant difference in the polysaccharide and protein contents between the different treatment groups (*p* > 0.05). There was no significant linear relationship between the voltage and the polysaccharide and protein contents in the dried samples. The protein content of shiitake mushroom slices dried by 26 kV and 34 kV voltage was the highest. The polysaccharide content of shiitake mushroom slices dried by 22 kV voltage was the highest. The reason for this result may be that EHD drying greatly shortens the drying time, resulting in a shorter interval and lower intensity of mushroom respiration, which reduces the consumption of polysaccharides and proteins [65,66]. On the other hand, the nonthermal characteristics of EHD drying can avoid the Maillard reaction and caramelization reaction in the drying process and further preserve polysaccharides and proteins [10,67].

## 4. Conclusions

The drying rate and effective moisture diffusion coefficient of shiitake mushroom slices treated with different voltages were significantly higher than those of the control group, and they had a higher shrinkage and lower rehydration ratio. Shiitake mushroom slices dried by EHD had better color performance than the control group. SEM showed that the surface of shiitake mushroom slices dried by the EHD system exhibited different degrees of encrustation, and the encrustation area increased with increasing voltage. In FTIR spectral analysis, the samples dried by the EHD system and the control group had similar absorption peak positions, but the absorption peak intensity of the EHD treatment group was higher than that of the control group. Compared with the control group, the shiitake mushroom slices dried by the EHD system had a higher protein content and polysaccharide content.

## Figures and Tables

**Table 1:** Color changes in Shiitake mushroom slices before and after drying.

Voltage	$L^{*}$	$a^{*}$	$b^{*}$	$\Delta E^{*}$
Fresh	74.4459 ± 0.8753 ^cd^	1.8220 ± 0.3328 ^b^	9.5188 ± 0.7823 ^c^	-
Control	73.9650 ± 0.8159 ^d^	2.6462 ± 0.5723 ^a^	20.0700 ± 0.7206 ^a^	10.4784 ± 0.8033 ^a^
18 kV	74.8200 ± 0.8769 ^bc^	2.7293 ± 0.4139 ^a^	14.2907 ± 1.2436 ^b^	5.0579 ± 0.4181 ^b^
22 kV	75.4375 ± 0.7744 ^ab^	2.7488 ± 0.4005 ^a^	13.7353 ± 1.4302 ^b^	4.7809 ± 0.3643 ^b^
26 kV	75.6675 ± 0.6802 ^a^	2.7733 ± 0.3682 ^a^	13.9467 ± 0.9326 ^b^	4.7748 ± 0.4011 ^b^
30 kV	75.7830 ± 0.5391 ^a^	2.8138 ± 0.3344 ^a^	12.8333 ± 1.1819 ^b^	4.5928 ± 0.4544 ^b^
34 kV	75.7000 ± 0.5956 ^a^	2.8225 ± 0.4744 ^a^	13.8836 ± 1.5334 ^b^	4.7255 ± 0.5278 ^b^

Note: Different letters indicate significant differences (*p* < 0.05) between sample means.

## Data Availability

Data on this study are available in the article.
